# Tranexamic acid use in a patient with sickle cell disease undergoing posterior scoliosis correction surgery: safely mitigating bleeding and vaso-occlusive crises

**DOI:** 10.1093/jscr/rjaa559

**Published:** 2021-01-29

**Authors:** Millie Newall, Thamer A Hamdan, Bisola Ajayi, Simon Weil, Timothy Bishop, Darren F Lui

**Affiliations:** St George’s University Hospital NHS Foundation Trust, London; St George’s University Hospital NHS Foundation Trust, London; St George’s University Hospital NHS Foundation Trust, London; St George’s University Hospital NHS Foundation Trust, London; St George’s University Hospital NHS Foundation Trust, London; St George’s University Hospital NHS Foundation Trust, London

**Keywords:** tranexamic acid, scoliosis, surgical correction, sickle cell disease

## Abstract

A 15-year-old female with 2-year post-menarchal adolescent idiopathic scoliosis and sickle cell disease (SCD) underwent posterior scoliosis correction surgery. SCD is associated with higher rates of surgical complications, and these patients require careful management to prevent vaso-occlusive sickle cell crises (VOSCC); scoliosis correction surgery can be associated with high morbidity and mortality, including significant blood loss.

Multiple techniques were employed to successfully prevent VOSCC in this patient including a preoperative transfusion, meticulous haemostasis at osteotomy sites, not performing a costoplasty despite presence of a rib hump, maintenance of intraoperative mean arterial pressure below 70 mmHg, aggressive postoperative hydration and the use of intraoperative tranexamic acid (TXA). This is the first reported case of the use of TXA in a patient with SCD and scoliosis correction surgery.

A satisfactory correction was achieved with a longer than average inpatient stay due to non-sickle cell pain and protracted wound ooze.

## INTRODUCTION

Scoliosis refers to a curve ≥10° observed clinically and with radiography. Idiopathic scoliosis accounts for around 80% of structural coronal scoliosis. Scoliosis affects females and males in a ratio of 1.4:1 for curves ≥10**°**, and 5:1 for curves >30**°** [1]. Progression is defined by a 5–10**°** increase in the curve and is expected with growth. Risser’s sign and Sanders classification of phalangeal physes indicate skeletal maturity. Surgical correction is generally indicated when the curve is >50**°** [2].

Sickle cell disease (SCD) is an autosomal recessive inherited haemoglobinopathy caused by a point mutation in the β-globin haemoglobin chain. The spine is commonly affected by acute SCD sequelae, including vaso-occlusive crises (VOSCC), osteomyelitis, spinal abscess, vertebral collapse and marrow necrosis, as well as chronic processes like avascular necrosis, arthritis, osteopenia and osteoporosis [3]. Biconcave ‘codfish’ vertebral deformities are common, but scoliosis is not typically associated with SCD.

SCD is associated with an increased risk of surgical complications [4], including blood loss leading to VOSCC. Tranexamic acid (TXA) was used in this case report to reduce bleeding and the need for post-operative transfusions.

**Figure 1:**
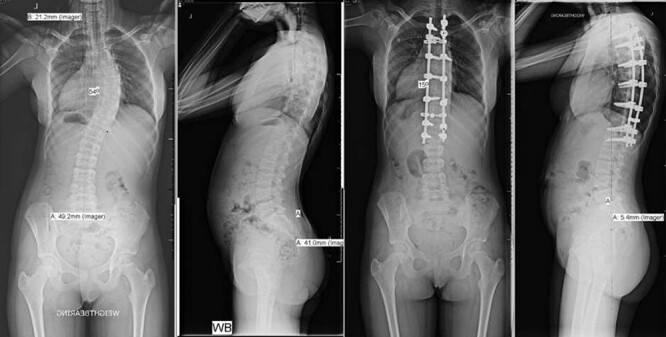
Pre- and post-operative spinal X-rays demonstrating 54° scoliosis curve, corrected with posterior fusion from T3 to L1.

TXA is a synthetic lysine derivative, which acts as an antifibrinolytic by binding plasminogen and preventing plasmin-mediated breakdown of fibrin clots; it is well established as an efficacious haemostatic agent [5].

## CASE HISTORY

A 15-year-old female with SCD underwent posterior scoliosis correction. One year prior to the surgery, she presented with two-year post-menarchal adolescent idiopathic scoliosis with right-sided trunk shift and rib hump, which was not fully reduced using the Adam’s forward bend test with right-side bending. There were no neurological signs or symptoms and no pain or functional impairment. X-ray showed that she was Risser stage 0 and had a right-sided mid-thoracic 40**°** curve from T4 to T11, which was maximal at T7, which progressed to 50**°** over 6 months. MRI showed biconcave ‘codfish’ vertebral deformities.

SCD was diagnosed as a newborn and she has had several VOSCC episodes, and one admission to paediatric intensive care unit (PICU) for acute chest syndrome at age seven. Relevant history includes vitamin D deficiency. She takes folic acid and prophylactic penicillin V and has annual influenza vaccinations.

An exchange transfusion was performed 1 week preoperatively, which reduced the percentage of sickled haemoglobin from 88 to 24% and increased the total haemoglobin from 80 to 109 g/L.

She underwent a posterior spinal fusion from T3 to L1 utilizing all pedicle screw construction. Total intravenous anaesthetic was employed to allow use of intraoperative trans-cranial spinal cord monitoring. A Misonix ultrasonic bone cutter was used for the facetectomies, and two diathermy generators were available for bipolar and monopolar use. Only one suction instrument was used and no cell saver. Generous Haemostat Surgiflo was used in each pedicle following instrumentation, and Spongostan foam, Surgicel and bone wax at the osteotomy sites. To further reduce bleeding, the mean arterial pressure (MAP) was maintained <70 mmHg and a costoplasty was avoided despite the rib hump. The total operation time was 220 min with 300 mL estimated blood loss (Fig. 1).

Our spinal surgery protocol includes crystalloid fluid and 10–15 mg per kg of TXA at induction with a further dose considered after 4 hours or if there is significant bleeding. Intraoperatively, this patient received 2.5 L of crystalloid fluid, a single 10 mg per kg intravenous TXA dose and prophylactic antibiotics.

Postoperatively, she was transferred to PICU with morphine patient-controlled analgesia. The following day she was moved to a paediatric ward, the analgesia was switched to oral tramadol, gabapentin and paracetamol and she began to eat, drink and mobilize.

The SCD was managed with incentive spirometry and aggressive intravenous hydration to prevent VOSCC, with a plan to transfuse if the haemoglobin was <70 g/L or if she needed supplemental oxygen, but this was not required.

She was discharged home eight days postoperatively with oral dihydrocodeine. Her recovery was satisfactory, although her stay was longer than average due to four days of non-infective oozing from the wound and non-SCD abdominal pain. At the eight-week review, the wound was well healed, a good clinical correction was achieved, and she was able to touch her knees. She reported residual left shoulder pain, which she managed with ibuprofen.

## DISCUSSION

Severe scoliosis may be treated with posterior spinal fusion surgery, particularly in the growing spine due to the risk of progression. Spinal fusion surgery in patients with SCD is associated with greater morbidity including wound and respiratory complications, pulmonary emboli and longer hospital stays [4]. SCD spine involvement can further complicate scoliosis surgery; immunocompromise can cause infections, vertebrae can collapse due to infarction, and osteoporosis and osteopenia [3] can lead to poor purchase of the transpedicular screw leading to risk of screw avulsion [6] and implant failure.

Scoliosis surgery can be associated with significant blood loss, which can cause VOSCC in SCD. Crises were avoided with a preoperative exchange transfusion, aggressive intravenous hydration and careful monitoring. We minimized bleeding with use of an ultrasonic bone cutter, limiting one suction device, keeping the MAP <70 mmHg and by giving TXA.

TXA reduces bleeding in spinal surgery [5] and can be given intravenously, locally or in combination. The potential side effects are nausea, vomiting, diarrhoea, arthralgia, myalgia, cramps, headache, migraine and fatigue. Seizures following application to the central nervous system have been recorded in animal studies [7]. TXA was associated with a threefold increase in venous thromboembolism in post-traumatic patients [8], which may be of particular concern in SCD; however, TXA was not associated with an increased risk of thrombosis in spinal surgery [9] and its use has been reported in patients with SCD for haematuria with a positive outcome [10]. No such complications were identified in this case, and a solid fixation and satisfactory correction were achieved with no requirement for postoperative transfusions.

In conclusion, we believe this is the first report of TXA use in patients with SCD undergoing scoliosis correction surgery. We advocate the use of intraoperative TXA in these patients to reduce bleeding and the risk of VOSCC. Further prospective studies should be considered.

## CONFLICT OF INTEREST STATEMENT

None declared.

## FUNDING

None.
